# Hémangiome capillaire de la paupière supérieure

**DOI:** 10.11604/pamj.2014.17.175.4096

**Published:** 2014-03-07

**Authors:** Hakima Elouarradi, Rajae Daoudi

**Affiliations:** 1 Université Mohammed V Souissi, Service d'Ophtalmologie A de l'Hôpital des Spécialités, Centre Hospitalier Universitaire, Rabat, Maroc

**Keywords:** Hémangiome, paupière, tuméfaction, œil, hemangioma, eyelid, swelling, eye

## Image en medicine

Enfant âgée de 1 ans, sans antécédents, emmenée par ses parents pour tuméfaction de la paupière supérieure droite apparaissant à l’âge de 2 mois et augmentant progressivement de taille. L'examen clinique objective une masse, de couleur violette de la paupière supérieure droite empiétant sur l'axe visuel (A). L'examen du segment antérieur et postérieur est normal. Patiente a bénéficié d'injections intra lésionnelles répétées de corticothérapie. Une nette diminution de la taille de l'hémangiome a été notée dès la 2ème injection intra lésionnelle de corticoïdes (B et C). Le suivi de l'enfant et le traitement est toujours en cours. Avec un recul de 1 ans, aucune complication liée à la corticothérapie ni à l'injection n'a été relevée. Les hémangiomes capillaires orbito-palpébraux sont des malformations vasculaires liées à une prolifération capillaire. Ils atteignent 10 à 12% des enfants. L'atteinte palpébrale est particulière du fait de l'anatomie spécifique des paupières et leurs rapports intimes avec l’œil. La décision thérapeutique est dictée par l’évolution spontanée ou sous traitement de la lésion, et par le risque d'amblyopie en cas d'occlusion palpébrale. Plusieurs méthodes ont été décrites dans la littérature: radiothérapie, laser, corticoïdes par voie locale en injection intra lésionnelle et générale, bétabloquants per os en cas de cortico résistance ou même chirurgie classique. Chaque méthode a une efficacité variable ainsi que des effets secondaires connus. La chirurgie doit être réservée en cas d'absence de réponse aux corticoïdes intra tumoraux et de régression spontanée.

**Figure 1:**
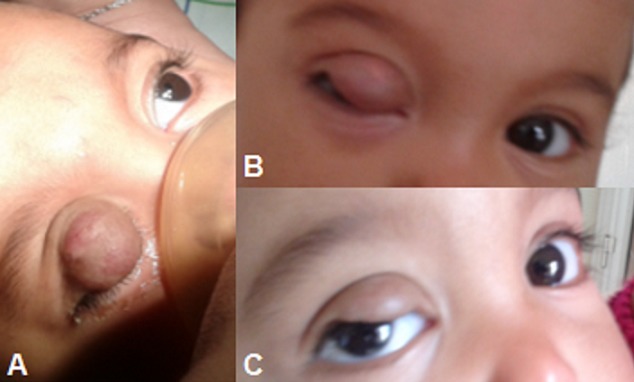
A) hémangiome capillaire de la paupière supérieure droite avant l'injection intra lésionnelle de corticoïdes au cours d'un examen sous anesthésie générale; B) et C) Nette diminution de la taille de l'hémangiome après l'injection avec axe visuel qui s'est dégagé

